# The Book World of Medicine and Science

**Published:** 1903-06-27

**Authors:** 


### The Book World of Medicine and Science.

Cancer of the Uterus: A Clinical Monograph. By A. H. Lewers, M.D., F.R.C.P. (London: H. K. Lewis. Pp. 382. 8vo. 1902.)

Dr. Lewers' monograph is ambitious, but we do not say his ambition is altogether unfulfilled. In the book the subject is treated almost entirely from the clinical point of view, and the fact is brought into prominence that if cancer of the uterus be met with in a reasonably early stage there is very good prospect that operative treatment may secure permanent relief. Nineteen cases are brought forward, 14 of cancer of the cervix, and five of the body of the uterus, in which periods of from four to 15 years have elapsed since the operation without return of the disease. Evidence is adduced in each case that the disease was really cancer, and details are given of the evidence on which it is claimed the disease has not recurred. Of course, these 19 cases are only a fraction of those observed by Dr. Lewers—perhaps 2 or 3 per cent. of the total. But Dr. Lewers thinks that if many of these other cases had been observed earlier benefits might for them have been secured by operation. Early diagnosis, then, is what is required if uterine cancer is to be succcessfully treated. Dr. Lewers throws out early in the work a suggestion which might, he thinks, be beneficially adopted by some corporate body such as a Joint Committee of the Royal Colleges of Surgeons and Physicians. The suggestion is that a leaflet should be drawn up, mentioning the facts which it is desirable for women to know—the possible significance of irregular bleeding for instance—and that this leaflet be distributed by medical men to matrons, nurses, district visitors, and so forth. Whatever may be thought of the practical utility of such a plan, of course everyone will sympathise with its intention. To turn to the subject matter proper of the book, we note that Dr. Lewers uses the term cancer as synonymous with the expression "malignant disease." Cancer therefore includes carcinoma sarcoma, and "some of the adenomata." With regard to ætiology, Dr. Lewers gives figures to support the view that patients who have cancer of the cervix have been more fertile than women not the subjects of the disease. But these figures are not conclusive. Dr. Lewers shows merely that amongst 100 women with advanced cancer of the cervix, more multiparæ were found than amongst 100 women not the subjects of cancer. But the non-cancerous hundred women were not healthy women, they were private patients of Dr. Lewers and perhaps pathologically sterile. Heredity, Dr. Lewers thinks, plays no important part in predisposing to cancer. There are no further remarks on ætiology. But then in this book no pretence of pathological investigation is made. It is a "clinical monograph." If no very new matter is brought forward, yet the book is interesting as the expression of the experience of a successful operator.

The Medical Annual for 1903. (Bristol: John Wright and Co. Price 7s. 6d. Pages 816.)

For many years past we have had an opportunity of recording in these columns the progressive evolution of this annual. The present volume marks an important event in the history of the publication, namely, its twenty-first year and coming of age. In honour of the occasion certain innovations have been introduced which will, we imagine, add considerably to the value of this and succeeding issues, and reflect even more accurately than heretofore the general progress of medical science. The chief innovation to which we refer is the addition of a general summary of the year's work, so that a comprehensive idea of the more important advances in medical progress may be more quickly attained. In this section of the work an important article is contributed by Mr. John Macintyre on the subject of $x$-rays, high-frequency currents and light treatment. This article is profusely illustrated with diagrams and photographic reproductions. The whole of the recent literature of electro-therapeutics is amply reviewed and brought up to date. The general review of medical and surgical progress has been compiled by a number of contributors, many of whom may be considered among the first authorities on their special subjects, among the more important articles being that of Dr. James Kerr Love on "Diseases of the Ear," and another on "Congenital Dislocation of the Hip," by Mr. Keith Monsarrat. The article on "infant feeding," by Dr. Henry Dwight Chapin, though affording an excellent account of his own special method, can hardly be considered as fully representing more recent progress in this department of dietetics. Taken as a whole, the present volume must be regarded as one of the best, and one of the most complete numbers which has as yet appeared of the "Medical Annual."

Indigestion: Its Prevention and Cure. By T. Herbert Alderson, M.B. (Walter Scott Publishing Co., London and Newcastle-upon-Tyne. Pages 130. Price 1s).

According to the description on the title-page this little volume is "a handy book of reference to the advice received in the physician's consulting-room." For all we know to the contrary, this description may be perfectly accurate, but it is quite beyond our powers of understanding to comprehend not only the above sentence, but also a very large number of the ragged and tattered strings of words which in almost unbroken continuity essay to describe how indigestion is prevented and cured. We cannot refrain from quoting a few of these gems of phraseology. Can any reader of The Hospital offer any suggestion as to the meaning, for instance, of the following:—"Plum-duff, roly-poly, marmalade, ginger, canary pudding, the glory of our youth home for the holidays, do you remember? Not now! If in perfect health and at midday laying in store of fuel for bicycling, shooting, football, tennis, golfing, croquet, who will say 'nay'? Not I." Or again "True, bread and cheese with an onion, form an ideal diet and contain all the requisites of a perfect food, and is a common meal with the labourer in health, but not for the citizen or the countryman with a sore stomach." And, again, "Habit is a great corrector of and a guide against constipation," and "So that nothing more difficult than a glass of water with (or without, as may be found necessary) two or three teaspoons full of sulphate of magnesia or sulphate of soda dissolved in the water taken immediately on rising, is in many cases all that is required to correct constipation, and to cure all its evil consequences"; and, finally, "as regards dessert, if on the table it has been pretty to look at and is useful in that way." It is impossible to write a serious review of a book which is composed of literary material of this kind. To those who propose to read this remarkable semi-medical production for the sake of supplementing the advice they receive in the consulting-room, we can only offer the same suggestion that Mr. *Punch* offered to those about to marry, namely, "Don't." On the other hand we would not, by reason of its literary and medical shortcomings, deprive readers of half an hour's amusement if they propose to read Dr. Alderson's book with this object in view.

